# Personal

**Published:** 1905-06

**Authors:** 


### PERSONAL.

Dr. Catherine E. Kelly, of Buffalo, has removed her offices from 615 Elmwood avenue to 33 Bremen street.

Dr. James A. Gibson, of Buffalo, has removed his residence from the Touraine to 170 Mariner street. His office is removed from 50 West Tupper street to his residence above noted.

Dr. L. Pierce Clark, of New York, has been appointed consulting neurologist at the Craig Colony for Epileptics, at Sonyea.

Dr. Max C. Breuer, of Buffalo, will sail for Europe, June 26, 1905, for a four months' tour in Germany. He will be accompanied by his family.

Dr. E. L. Shurly, of Detroit, was the guest of Dr. DeLancey Rochester during university commencement week. Dr. Shurly read a paper entitled, "Concerning the Etiology of Appendicitis," before the alumni association, which will appear in a future issue of the Journal.

Dr. Jacob S. Otto, of Buffalo, announces the change of his residence and office from 90 North Pearl street to 121 North Pearl street.

Dr. F. Park Lewis, of Buffalo, has been appointed by Governor Higgins a trustee of the New York State Institution for the Blind at Batavia. Dr. Lewis at present is serving as president of the board of managers.

Dr. Eugene H. Porter, of New York, has been appointed state commissioner of health vice Dr. Daniel Lewis, whose term has expired.

Dr. F. E. Hill, of Buffalo, has removed from 511 Swan street to 545 South Division street. Both telephones. Hours: 1 to 2 and 7 to 8 p. m.

Dr. Joel Henry Greene, a graduate of the University of Buffalo, 1875, of Dubuque, Iowa, attended the alumni association meeting last month and was elected president of his class. Dr. Greene expressed himself as greatly pleased with his visit to Buffalo.

---

Dr. Harry M. Weed, of Buffalo, announces that he has taken the offices of the late Dr. Henry D. Ingraham, 405 Franklin street, Buffalo. Practice limited to eye, ear, nose and throat. Hours: 9 to 1 and by appointment.

---

Dr. Vertner Kenerson, of Buffalo, has removed his offices to 181 Allen street. Telephones: Bell, Tupper 70; Frontier, 70.

